# Supplementary material for: Polyketide synthase gene clusters in the ericoid mycorrhizal fungus Oidiodendron maius: genome-wide prediction, expression profiling, and functional characterization of knockout mutants
Source: Front Plant Sci. 2026 Sep 16;17:1924430. doi: 10.3389/fpls.2026.1924430 (PMC13623903; doi:10.3389/fpls.2026.1924430)
Supplement: Supplementary file 1 [file SupplementaryFile1.zip › Supplementary Materials/Supplementary Folder 1.zip!/Oidma1_AssemblyScaffolds_Repeatmasked/knownclusterblast/cluster55/jgi.p_Oidma1_173662_mibig_hits.html]

| MIBiG Protein | Description | MIBiG Cluster | MiBiG Product | % ID | % Coverage | BLAST Score | E-value |
| --- | --- | --- | --- | --- | --- | --- | --- |
| AAV66110.2 | fusaridione\_A\_synthetase | BGC0000992 | Fusaridione\_A\_biosynthetic\_gene\_cluster | 35 | 104.011065007 | 379 | 8.4e-105 |
| AGO86662.1 | equisetin\_synthetase | BGC0001255 | Equisetin\_biosynthetic\_gene\_cluster | 36 | 102.627939142 | 368 | 1.9e-101 |
| XP\_001220460.1 | hypothetical\_protein | BGC0001182 | Chaetoglobosins\_biosynthetic\_gene\_cluster | 34 | 100.276625173 | 324 | 2.4e-88 |
| CBF80487.1 | hybrid\_PKS-NRPS\_(Eurofung) | BGC0000959 | Aspyridone\_biosynthetic\_gene\_cluster | 34 | 100.138312586 | 315 | 1.9e-85 |
| AAT28740.1 | FUSS | BGC0000064 | Fusarin\_biosynthetic\_gene\_cluster | 34 | 98.3402489627 | 312 | 9.6e-85 |
| AFP73394.1 | FusA | BGC0001268 | Fusarin\_biosynthetic\_gene\_cluster | 35 | 89.0733056708 | 310 | 6.2e-84 |
| EAW09117.1 | hybrid\_NRPS/PKS\_enzyme,\_putative | BGC0000983 | Cytochalasin\_biosynthetic\_gene\_cluster | 33 | 99.7233748271 | 298 | 2.5e-80 |
| ACS68554.1 | hybrid\_PKS-NRPS\_protein | BGC0001026 | NG-391\_biosynthetic\_gene\_cluster | 31 | 100.0 | 291 | 2.3e-78 |
| EAL85113.2 | hybrid\_PKS-NRPS\_enzyme,\_putative | BGC0001037 | Pseurotin\_A\_biosynthetic\_gene\_cluster | 31 | 99.5850622407 | 283 | 6.3e-76 |
| EAU38971.1 | hypothetical\_protein | BGC0001122 | Isoflavipucine\_biosynthetic\_gene\_cluster | 31 | 101.383125864 | 273 | 6.5e-73 |
| AKC54422.1 | fumosorinone\_biosynthesis\_polyketide\_synthase | BGC0001218 | Fumosorinone\_biosynthetic\_gene\_cluster | 34 | 88.2434301521 | 253 | 9e-67 |
| ADN43685.1 | DmbS | BGC0001136 | Desmethylbassianin\_biosynthetic\_gene\_cluster | 33 | 89.0733056708 | 248 | 1.7e-65 |
| BAK26562.1 | PKS-NRPS\_hybrid | BGC0000977 | Cyclopiazonic\_acid\_biosynthetic\_gene\_cluster | 31 | 90.1798063624 | 244 | 4.2e-64 |
| CAO91861.1 | putative\_PKS-NRPS\_protein | BGC0000968 | Chaetoglobosin\_biosynthetic\_gene\_cluster | 28 | 105.117565698 | 227 | 4.1e-59 |
| CAL69597.1 | nonribosomal\_peptide\_synthase | BGC0001049 | Tenellin\_biosynthetic\_gene\_cluster | 31 | 90.3181189488 | 224 | 3.4e-58 |
| AAT12283.1 | LtxA | BGC0000384 | Lyngbyatoxin\_biosynthetic\_gene\_cluster | 30 | 95.9889349931 | 191 | 2.5e-48 |
| AGD80618.1 | non-ribosomal\_peptide\_synthetase | BGC0000394 | Naphthyridinomycin\_biosynthetic\_gene\_cluster | 29 | 97.0954356846 | 185 | 3e-46 |
| AHJ31215.1 | Long-chain-fatty-acid--CoA\_ligase | BGC0000430 | Spumigin\_biosynthetic\_gene\_cluster | 29 | 89.6265560166 | 179 | 1.3e-44 |
| ANQ43349.1 | non-ribosomal\_peptide\_synthetase | BGC0001347\_c2 | Poaeamide\_B\_biosynthetic\_gene\_cluster | 38 | 48.4094052559 | 174 | 4.1e-43 |
| AAO23334.1 | NcpB | BGC0000397 | Nostocyclopeptide\_biosynthetic\_gene\_cluster | 29 | 85.0622406639 | 173 | 9.1e-43 |
| AAC06348.1 | bacitracin\_synthetase\_3 | BGC0000310 | Bacitracin\_biosynthetic\_gene\_cluster | 35 | 49.1009681881 | 173 | 1.2e-42 |
| KPN90369.1 | NunE | BGC0001416 | Nunapeptin\_/\_nunamycin\_biosynthetic\_gene\_cluster | 32 | 55.1867219917 | 173 | 1.2e-42 |
| CAD17792.1 | probable\_non\_ribosomal\_peptide\_synthetase\_protein | BGC0001363 | Ralsolamycin\_biosynthetic\_gene\_cluster | 36 | 50.4840940526 | 172 | 1.6e-42 |
| AAG02349.1 | peptide\_synthetase\_NRPS11-10 | BGC0000963 | Bleomycin\_biosynthetic\_gene\_cluster | 36 | 50.3457814661 | 171 | 3.5e-42 |
| CBJ79916.1 | putative\_Ornithine\_racemase | BGC0001133 | Taxlllaid\_biosynthetic\_gene\_cluster | 36 | 48.8243430152 | 171 | 3.5e-42 |
| CAD17793.1 | probable\_non\_ribosomal\_peptide\_synthetase\_protein | BGC0001363 | Ralsolamycin\_biosynthetic\_gene\_cluster | 37 | 49.1009681881 | 171 | 4.5e-42 |
| CAJ34381.1 | NRPS\_protein | BGC0000445 | Thiocoraline\_biosynthetic\_gene\_cluster | 36 | 52.0055325035 | 171 | 4.5e-42 |
| AED90002.1 | non-ribosomal\_peptide\_synthetase\_ThaA | BGC0000443 | Thanamycin\_biosynthetic\_gene\_cluster | 35 | 49.377593361 | 170 | 7.7e-42 |
| BAO84866.1 | putative\_non-ribosomal\_peptide\_synthetase | BGC0000414 | Quinocarcin\_biosynthetic\_gene\_cluster | 29 | 98.0636237898 | 170 | 1e-41 |
| AFH75330.1 | nonribosomal\_peptide\_synthetase | BGC0000398 | Orfamide\_biosynthetic\_gene\_cluster | 37 | 48.2710926694 | 170 | 1e-41 |
| AKC91849.1 | nonribosomal\_peptide\_synthetase | BGC0001414 | Griselimycin\_biosynthetic\_gene\_cluster | 36 | 48.9626556017 | 168 | 2.9e-41 |
| CBJ79915.1 | putative\_Phenylalanine\_racemase\_(ATP-hydrolyzing) | BGC0001133 | Taxlllaid\_biosynthetic\_gene\_cluster | 37 | 45.3665283541 | 168 | 3.8e-41 |
| ACZ55944.1 | non-ribosomal\_peptide\_synthetase | BGC0000302 | Anabaenopeptin\_biosynthetic\_gene\_cluster | 35 | 49.1009681881 | 168 | 3.8e-41 |
| AHF21228.1 | TriD | BGC0000449 | Tridecaptin\_biosynthetic\_gene\_cluster | 35 | 44.9515905947 | 167 | 5e-41 |
| AGE11898.1 | nonribosomal\_peptide\_synthetase | BGC0000366 | Gobichelin\_biosynthetic\_gene\_cluster | 35 | 49.5159059474 | 167 | 5e-41 |
| AED90003.1 | non-ribosomal\_peptide\_synthetase\_ThaB | BGC0000443 | Thanamycin\_biosynthetic\_gene\_cluster | 35 | 49.377593361 | 167 | 5e-41 |
| AEH59099.1 | amino\_acid\_adenylation\_domain-containing\_protein/NRPS | BGC0000385 | Lysobactin\_biosynthetic\_gene\_cluster | 37 | 45.7814661134 | 167 | 6.5e-41 |
| AKL78825.1 | GLNRPS7 | BGC0001187 | Xenolozoyenone\_biosynthetic\_gene\_cluster | 26 | 97.9253112033 | 167 | 6.5e-41 |
| AOA33123.1 | nonribosomal\_peptide\_synthetase | BGC0001346 | Bananamides\_biosynthetic\_gene\_cluster | 36 | 48.5477178423 | 166 | 8.5e-41 |
| AEU11006.1 | NpnB | BGC0001029 | Nostophycin\_biosynthetic\_gene\_cluster | 35 | 47.0262793914 | 166 | 8.5e-41 |
| BAC67536.1 | arthrofactin\_synthetase\_C | BGC0000305 | Arthrofactin\_biosynthetic\_gene\_cluster | 37 | 46.887966805 | 166 | 1.1e-40 |
| AHZ34233.1 | CifB | BGC0000323 | Cichofactin\_biosynthetic\_gene\_cluster | 35 | 52.6970954357 | 166 | 1.1e-40 |
| ALD82526.1 | non-ribosomal\_peptide\_synthase | BGC0001212 | Nannocystin\_biosynthetic\_gene\_cluster | 35 | 52.5587828492 | 166 | 1.1e-40 |
| ABC87508.1 | NRPS\_for\_pipecolate\_incorporation | BGC0001011 | Meridamycin\_biosynthetic\_gene\_cluster | 34 | 49.1009681881 | 166 | 1.1e-40 |
| ctg1\_orf19 |  | BGC0001013 | Meridamycin\_biosynthetic\_gene\_cluster | 34 | 49.1009681881 | 166 | 1.1e-40 |
| CBW45648.1 | PI\_synthetase\_2 | BGC0000952 | Pristinamycin\_biosynthetic\_gene\_cluster | 36 | 47.5795297372 | 166 | 1.1e-40 |
| EWS95124.1 | hypothetical\_protein | BGC0000306 | Arylomycin\_biosynthetic\_gene\_cluster | 36 | 48.9626556017 | 165 | 1.9e-40 |
| AAY37647.1 | Amino\_acid\_adenylation | BGC0000437 | Syringomycin\_biosynthetic\_gene\_cluster | 38 | 44.6749654219 | 165 | 3.2e-40 |
| ABI26077.1 | OciA | BGC0000331 | Cyanopeptin\_biosynthetic\_gene\_cluster | 35 | 48.5477178423 | 163 | 7.2e-40 |
| CCJ67636.1 | TaaA | BGC0000447 | Tolaasin\_biosynthetic\_gene\_cluster | 37 | 49.5159059474 | 163 | 7.2e-40 |
| CAJ34382.1 | NRPS\_protein | BGC0000445 | Thiocoraline\_biosynthetic\_gene\_cluster | 29 | 84.6473029046 | 163 | 9.4e-40 |
| KPN93063.1 | NupA | BGC0001416 | Nunapeptin\_/\_nunamycin\_biosynthetic\_gene\_cluster | 37 | 47.7178423237 | 163 | 9.4e-40 |
| CCJ67640.1 | TaaE | BGC0000447 | Tolaasin\_biosynthetic\_gene\_cluster | 35 | 50.4840940526 | 163 | 9.4e-40 |
| ACM79812.1 | ZmaQ | BGC0001059 | Zwittermycin\_A\_biosynthetic\_gene\_cluster | 34 | 52.2821576763 | 163 | 9.4e-40 |
| AAF08795.1 | MycA | BGC0001103 | Mycosubtilin\_biosynthetic\_gene\_cluster | 36 | 50.2074688797 | 163 | 1.2e-39 |
| ABS75232.1 | DhbF | BGC0001185 | Bacillibactin\_biosynthetic\_gene\_cluster | 35 | 51.4522821577 | 162 | 1.6e-39 |
| BAH22763.1 | nonribosomal\_peptide\_synthetase | BGC0001018 | Micropeptin\_biosynthetic\_gene\_cluster | 34 | 49.6542185339 | 162 | 1.6e-39 |
| AHY86403.1 | non\_ribosomal\_peptide\_synthetase | BGC0000329 | Corpeptin\_biosynthetic\_gene\_cluster | 38 | 48.5477178423 | 162 | 1.6e-39 |
| AHZ34238.1 | CipA | BGC0001389 | Cichopeptin\_biosynthetic\_gene\_cluster | 37 | 48.4094052559 | 162 | 2.1e-39 |
| AAZ03551.1 | McnB | BGC0000332 | Cyanopeptolin\_biosynthetic\_gene\_cluster | 34 | 49.6542185339 | 161 | 2.7e-39 |
| AAO72425.1 | syringopeptin\_synthetase\_C | BGC0000438 | Syringopeptin\_biosynthetic\_gene\_cluster | 37 | 47.7178423237 | 161 | 2.7e-39 |
| KPN93065.1 | NunD | BGC0001416 | Nunapeptin\_/\_nunamycin\_biosynthetic\_gene\_cluster | 37 | 48.5477178423 | 161 | 2.7e-39 |
| ABJ97436.1 | MerP | BGC0001012 | Meridamycin\_biosynthetic\_gene\_cluster | 33 | 51.867219917 | 161 | 2.7e-39 |
| AGM14934.1 | xantholysin\_synthetase\_C | BGC0000463 | Xantholysin\_biosynthetic\_gene\_cluster | 36 | 50.0691562932 | 161 | 2.7e-39 |
| ACA97580.1 | PmxE | BGC0000408 | Polymyxin\_biosynthetic\_gene\_cluster | 39 | 44.8132780083 | 161 | 3.6e-39 |
| CAJ18237.2 | non-ribosomal\_peptide\_synthetase\_B | BGC0000354 | Friulimicin\_biosynthetic\_gene\_cluster | 36 | 46.1964038728 | 161 | 3.6e-39 |
| AAY37655.1 | Amino\_acid\_adenylation | BGC0000437 | Syringomycin\_biosynthetic\_gene\_cluster | 37 | 47.7178423237 | 161 | 3.6e-39 |
| CCA29203.1 | non-ribosomal\_peptide\_synthetase/polyketide\_synthase | BGC0000955 | Althiomycin\_biosynthetic\_gene\_cluster | 36 | 51.3139695712 | 161 | 3.6e-39 |
| AAX31559.1 | peptide\_synthetase\_3 | BGC0000336 | Daptomycin\_biosynthetic\_gene\_cluster | 31 | 63.4854771784 | 161 | 3.6e-39 |
| AEH59100.1 | amino\_acid\_adenylation\_domain-containing\_protein/NRPS | BGC0000385 | Lysobactin\_biosynthetic\_gene\_cluster | 34 | 56.846473029 | 161 | 3.6e-39 |
| AAX31558.1 | peptide\_synthetase\_2 | BGC0000336 | Daptomycin\_biosynthetic\_gene\_cluster | 31 | 67.6348547718 | 161 | 3.6e-39 |
| AEZ51520.1 | PmxE | BGC0001153 | Polymyxin\_biosynthetic\_gene\_cluster | 39 | 44.8132780083 | 161 | 4.7e-39 |
| CCP45168.1 | Peptide\_synthetase\_MbtE\_(peptide\_synthase) | BGC0001021 | Mycobactin\_biosynthetic\_gene\_cluster | 35 | 49.6542185339 | 161 | 4.7e-39 |
| AHZ34239.1 | CipB | BGC0001389 | Cichopeptin\_biosynthetic\_gene\_cluster | 36 | 49.9308437068 | 161 | 4.7e-39 |
| AGE25480.1 | amino\_acid\_adenylation\_protein | BGC0001208\_c2 | Poaeamide\_biosynthetic\_gene\_cluster | 36 | 48.4094052559 | 161 | 4.7e-39 |
| AAO23333.1 | NcpA | BGC0000397 | Nostocyclopeptide\_biosynthetic\_gene\_cluster | 34 | 47.5795297372 | 161 | 4.7e-39 |
| AAO72424.1 | syringopeptin\_synthetase\_B | BGC0000438 | Syringopeptin\_biosynthetic\_gene\_cluster | 37 | 45.0899031812 | 161 | 4.7e-39 |
| AOA33122.1 | nonribosomal\_peptide\_synthetase | BGC0001346 | Bananamides\_biosynthetic\_gene\_cluster | 36 | 51.0373443983 | 160 | 6.1e-39 |
| AJF34464.1 | Txo2 | BGC0001207 | Teixobactin\_biosynthetic\_gene\_cluster | 34 | 51.3139695712 | 160 | 6.1e-39 |
| AHD05677.1 | nonribosomal\_peptide\_ligase\_subunit | BGC0000402 | Paenilarvins\_biosynthetic\_gene\_cluster | 36 | 51.7289073306 | 160 | 8e-39 |
| AEH41794.1 | HrmP | BGC0000374 | Hormaomycin\_biosynthetic\_gene\_cluster | 33 | 53.5269709544 | 160 | 8e-39 |
| AFJ23819.1 | WLIP\_synthetase\_A | BGC0000462 | WLIP\_biosynthetic\_gene\_cluster | 34 | 51.4522821577 | 160 | 8e-39 |
| ABV79987.1 | ApnC | BGC0000301 | Anabaenopeptin\_biosynthetic\_gene\_cluster | 32 | 50.0691562932 | 160 | 8e-39 |
| AKC91856.1 | nonribosomal\_peptide\_synthetase | BGC0001414 | Griselimycin\_biosynthetic\_gene\_cluster | 35 | 47.8561549101 | 160 | 1e-38 |
| AEU11003.1 | NpnC | BGC0001029 | Nostophycin\_biosynthetic\_gene\_cluster | 35 | 44.2600276625 | 160 | 1e-38 |
| CDF96614.1 | Long-chain-fatty-acid--CoA\_ligase | BGC0001149 | Brabantamide\_biosynthetic\_gene\_cluster | 35 | 53.112033195 | 159 | 1.4e-38 |
| AAF17280.1 | NosC | BGC0001028 | Nostopeptolide\_biosynthetic\_gene\_cluster | 33 | 49.6542185339 | 159 | 1.8e-38 |
| CAC11137.1 | NikP1\_protein | BGC0000876 | Nikkomycin\_biosynthetic\_gene\_cluster | 35 | 49.2392807746 | 158 | 2.3e-38 |
| AEA30274.1 | peptide\_synthetase | BGC0000429 | Skyllamycin\_biosynthetic\_gene\_cluster | 34 | 46.1964038728 | 158 | 2.3e-38 |
| ACZ55946.1 | non-ribosomal\_peptide\_synthetase | BGC0000302 | Anabaenopeptin\_biosynthetic\_gene\_cluster | 36 | 44.398340249 | 158 | 2.3e-38 |
| CDG17985.1 | Putative\_Ornithine\_racemase\_(fragment) | BGC0000464 | Xenoamicins\_biosynthetic\_gene\_cluster | 34 | 50.7607192254 | 158 | 2.3e-38 |
| KFH48607.1 | N-like\_protein | BGC0000317 | Cephalosporin\_biosynthetic\_gene\_cluster | 36 | 46.887966805 | 158 | 3e-38 |
| AJF34463.1 | Txo1 | BGC0001207 | Teixobactin\_biosynthetic\_gene\_cluster | 34 | 49.9308437068 | 158 | 4e-38 |
| CDG17987.1 | Putative\_Ornithine\_racemase\_(fragment) | BGC0000464 | Xenoamicins\_biosynthetic\_gene\_cluster | 34 | 51.867219917 | 157 | 5.2e-38 |
| CAA11795.1 | PCZA363.4 | BGC0000322 | Chloroeremomycin\_biosynthetic\_gene\_cluster | 37 | 45.7814661134 | 157 | 5.2e-38 |
| CAM56770.1 |  | BGC0000354 | Friulimicin\_biosynthetic\_gene\_cluster | 35 | 49.377593361 | 157 | 5.2e-38 |
| AAF15891.2 | NosA | BGC0001028 | Nostopeptolide\_biosynthetic\_gene\_cluster | 35 | 51.1756569848 | 157 | 5.2e-38 |
| AFH75322.1 | nonribosomal\_peptide\_synthetase | BGC0000425 | Sessilin\_biosynthetic\_gene\_cluster | 34 | 48.9626556017 | 157 | 5.2e-38 |
| ADA69239.2 | trans-AT\_hybrid\_polyketide\_synthase-NRPS | BGC0001071 | Nosperin\_biosynthetic\_gene\_cluster | 35 | 48.132780083 | 157 | 5.2e-38 |
| AGM16413.1 | paenibacterin\_synthetase\_B | BGC0000400 | Paenibacterin\_biosynthetic\_gene\_cluster | 34 | 45.9197786999 | 157 | 6.8e-38 |
| AHD05618.1 | putative\_non-ribosomal\_peptide\_ligase\_domain\_protein | BGC0001033 | Paenilamicin\_biosynthetic\_gene\_cluster | 35 | 46.1964038728 | 157 | 6.8e-38 |
| AAU39359.1 | lichenysin\_synthase\_LchAA | BGC0000381 | Lichenysin\_biosynthetic\_gene\_cluster | 36 | 56.0165975104 | 157 | 6.8e-38 |
| CDG17981.1 | Non-ribosomal\_peptide\_synthetase | BGC0000464 | Xenoamicins\_biosynthetic\_gene\_cluster | 36 | 49.377593361 | 157 | 6.8e-38 |
| AGM16412.1 | paenibacterin\_synthetase\_A | BGC0000400 | Paenibacterin\_biosynthetic\_gene\_cluster | 34 | 48.6860304288 | 156 | 1.2e-37 |
| AFP87549.1 | NrpS | BGC0001135 | Bicornutin\_biosynthetic\_gene\_cluster | 35 | 44.9515905947 | 156 | 1.5e-37 |
| CAQ34921.1 | nonribosomal\_peptide\_synthetase | BGC0000986 | Dkxanthene\_biosynthetic\_gene\_cluster | 34 | 51.867219917 | 156 | 1.5e-37 |
| ALV86866.1 | Tlo20 | BGC0001406 | Telomycin\_biosynthetic\_gene\_cluster | 36 | 50.3457814661 | 155 | 2e-37 |
| BAH43766.1 | tyrocidine\_synthetase\_III | BGC0000452 | Tyrocidine\_biosynthetic\_gene\_cluster | 36 | 44.6749654219 | 155 | 2e-37 |
| BAH43870.1 | putative\_linear\_pentadecapeptide\_gramicidin\_synthetase\_LgrB | BGC0000367 | Gramicidin\_biosynthetic\_gene\_cluster | 33 | 52.1438450899 | 155 | 2e-37 |
| CAL69889.1 | RhiB\_protein | BGC0001112 | Rhizoxins\_biosynthetic\_gene\_cluster | 33 | 46.0580912863 | 155 | 2.6e-37 |
| AAX31557.1 | peptide\_synthetase\_1 | BGC0000336 | Daptomycin\_biosynthetic\_gene\_cluster | 37 | 45.5048409405 | 155 | 3.4e-37 |
| CAK15815.1 | putative\_non\_ribosomal\_peptide\_synthetase | BGC0000344 | Entolysin\_biosynthetic\_gene\_cluster | 35 | 46.6113416321 | 155 | 3.4e-37 |
| ADN26248.1 | peptide\_synthetase | BGC0000951 | Pacidamycin\_biosynthetic\_gene\_cluster | 34 | 49.377593361 | 155 | 3.4e-37 |
| AGZ15458.1 | putative\_non-ribosomal\_peptide\_synthetase | BGC0001036 | Polyoxypeptin\_biosynthetic\_gene\_cluster | 34 | 49.7925311203 | 154 | 4.4e-37 |
| AAF08796.1 | MycB | BGC0001103 | Mycosubtilin\_biosynthetic\_gene\_cluster | 32 | 51.867219917 | 154 | 4.4e-37 |
| ABS74205.1 | FenE | BGC0001095 | Fengycin\_biosynthetic\_gene\_cluster | 34 | 47.3029045643 | 154 | 4.4e-37 |
| ABV79985.1 | ApnA | BGC0000301 | Anabaenopeptin\_biosynthetic\_gene\_cluster | 33 | 48.8243430152 | 154 | 5.7e-37 |
| AFH75329.1 | nonribosomal\_peptide\_synthetase | BGC0000398 | Orfamide\_biosynthetic\_gene\_cluster | 34 | 52.6970954357 | 153 | 7.5e-37 |
| CBD77749.1 | non-ribosomal\_peptide\_synthetase | BGC0000974 | Crocacin\_biosynthetic\_gene\_cluster | 32 | 61.5491009682 | 153 | 7.5e-37 |
| ABI26079.1 | OciC | BGC0000331 | Cyanopeptin\_biosynthetic\_gene\_cluster | 33 | 49.9308437068 | 153 | 7.5e-37 |
| KPN93064.1 | NupB | BGC0001416 | Nunapeptin\_/\_nunamycin\_biosynthetic\_gene\_cluster | 36 | 47.5795297372 | 153 | 7.5e-37 |
| AEZ51517.1 | PmxB | BGC0001153 | Polymyxin\_biosynthetic\_gene\_cluster | 34 | 44.6749654219 | 153 | 7.5e-37 |
| AIW82284.1 | PuwG | BGC0001125 | Puwainaphycins\_biosynthetic\_gene\_cluster | 34 | 48.9626556017 | 153 | 7.5e-37 |
| ACZ55942.1 | non-ribosomal\_peptide\_synthetase | BGC0000302 | Anabaenopeptin\_biosynthetic\_gene\_cluster | 36 | 44.9515905947 | 153 | 9.8e-37 |
| CBD77746.1 | non-ribosomal\_peptide\_synthetase/polyketide\_synthase | BGC0000974 | Crocacin\_biosynthetic\_gene\_cluster | 35 | 48.8243430152 | 153 | 9.8e-37 |
| AJM89738.1 | PmxE | BGC0001192 | Colistin\_biosynthetic\_gene\_cluster | 37 | 44.9515905947 | 153 | 9.8e-37 |
| AHZ34232.1 | CifA | BGC0000323 | Cichofactin\_biosynthetic\_gene\_cluster | 35 | 47.3029045643 | 153 | 9.8e-37 |
| AAF99707.2 | syringopeptin\_synthetase | BGC0000438 | Syringopeptin\_biosynthetic\_gene\_cluster | 36 | 48.8243430152 | 153 | 9.8e-37 |
| CCJ67648.1 | JagD | BGC0001127 | Jagaricin\_biosynthetic\_gene\_cluster | 34 | 49.1009681881 | 153 | 1.3e-36 |
| AAU34203.1 | mannopeptimycin\_peptide\_synthetase\_MppB | BGC0000388 | Mannopeptimycin\_biosynthetic\_gene\_cluster | 38 | 41.0788381743 | 153 | 1.3e-36 |
| AAZ03550.1 | McnA | BGC0000332 | Cyanopeptolin\_biosynthetic\_gene\_cluster | 31 | 51.5905947441 | 153 | 1.3e-36 |
| ACA97577.1 | PmxB | BGC0000408 | Polymyxin\_biosynthetic\_gene\_cluster | 34 | 46.3347164592 | 153 | 1.3e-36 |
| CAM56771.1 |  | BGC0000354 | Friulimicin\_biosynthetic\_gene\_cluster | 31 | 60.7192254495 | 153 | 1.3e-36 |
| AAU39360.1 | lichenysin\_synthase\_LchAB | BGC0000381 | Lichenysin\_biosynthetic\_gene\_cluster | 33 | 46.4730290456 | 152 | 1.7e-36 |
| AIW82283.1 | PuwF | BGC0001125 | Puwainaphycins\_biosynthetic\_gene\_cluster | 36 | 44.6749654219 | 152 | 1.7e-36 |
| ACY06285.1 | non-ribosomal\_peptide\_synthetase | BGC0001042 | Sanglifehrin\_A\_biosynthetic\_gene\_cluster | 34 | 48.132780083 | 152 | 1.7e-36 |
| BAC67535.1 | arthrofactin\_synthetase\_B | BGC0000305 | Arthrofactin\_biosynthetic\_gene\_cluster | 37 | 45.0899031812 | 152 | 2.2e-36 |
| KPN90376.1 | NupC | BGC0001416 | Nunapeptin\_/\_nunamycin\_biosynthetic\_gene\_cluster | 34 | 47.5795297372 | 152 | 2.2e-36 |
| AAO56329.1 | non-ribosomal\_peptide\_synthetase\_SyfB | BGC0000435 | Syringafactin\_biosynthetic\_gene\_cluster | 35 | 48.8243430152 | 152 | 2.2e-36 |
| CAE15637.1 |  | BGC0001128 | Luminmide\_biosynthetic\_gene\_cluster | 35 | 48.8243430152 | 151 | 2.8e-36 |
| CDG17982.1 | Non-ribosomal\_peptide\_synthetase | BGC0000464 | Xenoamicins\_biosynthetic\_gene\_cluster | 34 | 54.4951590595 | 151 | 2.8e-36 |
| AHZ20784.1 | non-ribosomal\_peptide\_synthase | BGC0000369 | Hassallidins\_biosynthetic\_gene\_cluster | 32 | 51.867219917 | 151 | 2.8e-36 |
| ALV86867.1 | Tlo21 | BGC0001406 | Telomycin\_biosynthetic\_gene\_cluster | 36 | 41.7704011065 | 151 | 2.8e-36 |
| CBJ90082.1 | Non\_Ribosomal\_peptide\_synthetase\_(-succinylbenzoate--CoA\_ligase) | BGC0001132 | Xenotetrapeptide\_biosynthetic\_gene\_cluster | 36 | 46.6113416321 | 151 | 2.8e-36 |
| CBW54672.1 | non\_ribosomal\_peptide\_synthetase | BGC0000971 | Cinnabaramide\_biosynthetic\_gene\_cluster | 33 | 50.4840940526 | 151 | 3.7e-36 |
| AGE11899.1 | nonribosomal\_peptide\_synthetase | BGC0000366 | Gobichelin\_biosynthetic\_gene\_cluster | 35 | 46.6113416321 | 151 | 4.8e-36 |
| ACZ55945.1 | non-ribosomal\_peptide\_synthetase | BGC0000302 | Anabaenopeptin\_biosynthetic\_gene\_cluster | 34 | 46.3347164592 | 151 | 4.8e-36 |
| AHB82069.1 | non\_ribosomal\_peptide\_synthetase | BGC0001231 | Microsclerodermins\_biosynthetic\_gene\_cluster | 33 | 48.9626556017 | 150 | 6.3e-36 |
| CAK15814.1 | putative\_non-ribosomal\_peptide\_synthetase,\_terminal\_component | BGC0000344 | Entolysin\_biosynthetic\_gene\_cluster | 34 | 50.2074688797 | 150 | 6.3e-36 |
| EFE73308.1 | non-ribosomal\_peptide\_synthetase | BGC0000431 | Stenothricin\_biosynthetic\_gene\_cluster | 34 | 46.7496542185 | 150 | 6.3e-36 |
| BAP16697.1 | nonribosomal\_peptide\_synthetase | BGC0000376 | JBIR-34,\_JBIR-35\_biosynthetic\_gene\_cluster | 30 | 53.9419087137 | 150 | 6.3e-36 |
| sln14 | nonribosomal\_peptide\_synthetase/polyketide\_synthase\_type\_I | BGC0001230 | Salinamides\_biosynthetic\_gene\_cluster | 36 | 51.3139695712 | 150 | 6.3e-36 |
| AIG79240.1 | Hypothetical\_protein | BGC0000419 | Ristomycin\_A\_biosynthetic\_gene\_cluster | 31 | 64.1770401107 | 150 | 8.3e-36 |
| ABF85931.1 | non-ribosomal\_peptide\_synthase/polyketide\_synthase\_Ta1 | BGC0001025 | Myxovirescin\_biosynthetic\_gene\_cluster | 33 | 48.9626556017 | 150 | 8.3e-36 |
| BAH43869.1 | linear\_pentadecapeptide\_gramicidin\_synthetase\_LgrA | BGC0000367 | Gramicidin\_biosynthetic\_gene\_cluster | 35 | 45.0899031812 | 150 | 8.3e-36 |
| AAY37654.1 | Amino\_acid\_adenylation | BGC0000437 | Syringomycin\_biosynthetic\_gene\_cluster | 35 | 48.9626556017 | 150 | 8.3e-36 |
| ABW17375.1 | PsoA | BGC0000411 | Putisolvin\_biosynthetic\_gene\_cluster | 32 | 50.622406639 | 150 | 8.3e-36 |
| ALG65313.1 | Cal23 | BGC0001297 | WS9326\_biosynthetic\_gene\_cluster | 34 | 45.2282157676 | 149 | 1.1e-35 |
| AGM14933.1 | xantholysin\_synthetase\_B | BGC0000463 | Xantholysin\_biosynthetic\_gene\_cluster | 37 | 42.1853388658 | 149 | 1.1e-35 |
| AAK81824.1 | peptide\_synthetase | BGC0000326 | Complestatin\_biosynthetic\_gene\_cluster | 35 | 43.9834024896 | 149 | 1.1e-35 |
| AAZ23075.1 | peptide\_synthetase | BGC0000291 | A54145\_biosynthetic\_gene\_cluster | 36 | 45.5048409405 | 149 | 1.4e-35 |
| AAG02355.1 | peptide\_synthetase\_NRPS9-8 | BGC0000963 | Bleomycin\_biosynthetic\_gene\_cluster | 35 | 50.2074688797 | 149 | 1.4e-35 |
| AIW82285.1 | PuwH | BGC0001125 | Puwainaphycins\_biosynthetic\_gene\_cluster | 33 | 48.4094052559 | 149 | 1.4e-35 |
| ABV79988.1 | ApnD | BGC0000301 | Anabaenopeptin\_biosynthetic\_gene\_cluster | 32 | 50.7607192254 | 149 | 1.4e-35 |
| AEZ51516.1 | PmxA | BGC0001153 | Polymyxin\_biosynthetic\_gene\_cluster | 35 | 49.377593361 | 149 | 1.8e-35 |
| ABS74208.1 | FenB | BGC0001095 | Fengycin\_biosynthetic\_gene\_cluster | 33 | 48.2710926694 | 148 | 2.4e-35 |
| AAW03330.1 | CtaG | BGC0000982 | Cystothiazole\_A\_biosynthetic\_gene\_cluster | 34 | 46.0580912863 | 148 | 2.4e-35 |
| ABS74180.1 | BmyB | BGC0001090 | Bacillomycin\_biosynthetic\_gene\_cluster | 33 | 48.8243430152 | 148 | 2.4e-35 |
| BAB69699.1 | iturin\_A\_synthetase\_B | BGC0001098 | Iturin\_biosynthetic\_gene\_cluster | 33 | 48.8243430152 | 148 | 2.4e-35 |
| CCJ67637.1 | TaaB | BGC0000447 | Tolaasin\_biosynthetic\_gene\_cluster | 35 | 49.2392807746 | 148 | 2.4e-35 |
| AEH41793.1 | HrmO | BGC0000374 | Hormaomycin\_biosynthetic\_gene\_cluster | 37 | 44.398340249 | 148 | 2.4e-35 |
| AHD05678.1 | nonribosomal\_peptide\_ligase\_subunit | BGC0000402 | Paenilarvins\_biosynthetic\_gene\_cluster | 38 | 45.643153527 | 148 | 3.1e-35 |
| AGM16414.1 | paenibacterin\_synthetase\_C | BGC0000400 | Paenibacterin\_biosynthetic\_gene\_cluster | 33 | 45.5048409405 | 148 | 3.1e-35 |
| sln8 | nonribosomal\_peptide\_synthetase | BGC0001230 | Salinamides\_biosynthetic\_gene\_cluster | 36 | 47.3029045643 | 148 | 3.1e-35 |
| AJM89735.1 | PmxA | BGC0001192 | Colistin\_biosynthetic\_gene\_cluster | 35 | 49.377593361 | 148 | 3.1e-35 |
| AAO56328.1 | non-ribosomal\_peptide\_synthetase\_SyfA | BGC0000435 | Syringafactin\_biosynthetic\_gene\_cluster | 35 | 50.2074688797 | 148 | 3.1e-35 |
| ABW17376.1 | PsoB | BGC0000411 | Putisolvin\_biosynthetic\_gene\_cluster | 32 | 60.0276625173 | 148 | 4.1e-35 |
| ADY76664.1 | non-ribosomal\_peptide\_synthetase | BGC0000950 | Napsamycin\_biosynthetic\_gene\_cluster | 36 | 47.0262793914 | 148 | 4.1e-35 |
| BAP16689.1 | nonribosomal\_peptide\_synthetase | BGC0000376 | JBIR-34,\_JBIR-35\_biosynthetic\_gene\_cluster | 34 | 47.0262793914 | 148 | 4.1e-35 |
| AIE77060.1 | peptide\_synthetase\_module\_7 | BGC0000418 | Ristocetin\_biosynthetic\_gene\_cluster | 31 | 64.1770401107 | 147 | 5.4e-35 |
| AOA33121.1 | nonribosomal\_peptide\_synthetase | BGC0001346 | Bananamides\_biosynthetic\_gene\_cluster | 37 | 45.0899031812 | 147 | 5.4e-35 |
| AFJ14793.1 | PlpD | BGC0000403 | Pelgipeptin\_biosynthetic\_gene\_cluster | 35 | 44.9515905947 | 147 | 5.4e-35 |
| BAH22765.1 | nonribosomal\_peptide\_synthetase | BGC0001018 | Micropeptin\_biosynthetic\_gene\_cluster | 31 | 52.0055325035 | 147 | 7e-35 |
| ACM79806.1 | ZmaB | BGC0001059 | Zwittermycin\_A\_biosynthetic\_gene\_cluster | 35 | 46.0580912863 | 146 | 9.1e-35 |
| AHZ20774.1 | non-ribosomal\_peptide\_synthase | BGC0000369 | Hassallidins\_biosynthetic\_gene\_cluster | 34 | 47.0262793914 | 146 | 9.1e-35 |
| AJW76709.1 | DsaG | BGC0001196 | Desotamide\_biosynthetic\_gene\_cluster | 34 | 49.1009681881 | 146 | 9.1e-35 |
| AAK81826.1 | peptide\_synthetase | BGC0000326 | Complestatin\_biosynthetic\_gene\_cluster | 35 | 44.5366528354 | 146 | 1.2e-34 |
| CDG17986.1 | Non-ribosomal\_peptide\_synthetase | BGC0000464 | Xenoamicins\_biosynthetic\_gene\_cluster | 34 | 48.6860304288 | 146 | 1.2e-34 |
| AEI58865.1 | peptide\_synthetase | BGC0000455 | Vancomycin\_biosynthetic\_gene\_cluster | 35 | 44.8132780083 | 146 | 1.2e-34 |
| AFH75321.1 | nonribosomal\_peptide\_synthetase | BGC0000425 | Sessilin\_biosynthetic\_gene\_cluster | 39 | 41.3554633472 | 146 | 1.6e-34 |
| sln7 | nonribosomal\_peptide\_synthetase | BGC0001230 | Salinamides\_biosynthetic\_gene\_cluster | 34 | 50.2074688797 | 146 | 1.6e-34 |
| CAD91211.1 | putative\_non-ribosomal\_peptide\_synthetase,\_module\_7 | BGC0000289 | A40926\_biosynthetic\_gene\_cluster | 33 | 56.5698478562 | 146 | 1.6e-34 |
| BAC67534.2 | arthrofactin\_synthetase\_A | BGC0000305 | Arthrofactin\_biosynthetic\_gene\_cluster | 33 | 56.4315352697 | 146 | 1.6e-34 |
| CAA11794.1 | PCZA363.3 | BGC0000322 | Chloroeremomycin\_biosynthetic\_gene\_cluster | 36 | 44.8132780083 | 145 | 2e-34 |
| AEW31022.1 | plipastatin\_synthetase | BGC0000407 | Plipastatin\_biosynthetic\_gene\_cluster | 32 | 49.9308437068 | 145 | 2.7e-34 |
| BAH22764.1 | nonribosomal\_peptide\_synthetase | BGC0001018 | Micropeptin\_biosynthetic\_gene\_cluster | 29 | 54.356846473 | 145 | 2.7e-34 |
| AFH75320.1 | nonribosomal\_peptide\_synthetase | BGC0000425 | Sessilin\_biosynthetic\_gene\_cluster | 33 | 58.5062240664 | 144 | 3.5e-34 |
| ACG60773.1 | NRPS(C/A/PCP) | BGC0001058 | Zorbamycin\_biosynthetic\_gene\_cluster | 35 | 45.5048409405 | 144 | 3.5e-34 |
| AIW82282.1 | PuwE | BGC0001125 | Puwainaphycins\_biosynthetic\_gene\_cluster | 37 | 41.2171507607 | 144 | 3.5e-34 |
| AAG34184.1 | SimH | BGC0000835 | Simocyclinone\_biosynthetic\_gene\_cluster | 33 | 47.1645919779 | 144 | 4.5e-34 |
| CAE53353.1 | non-ribosomal\_peptide\_synthetase | BGC0000440 | Teicoplanin\_biosynthetic\_gene\_cluster | 31 | 63.7621023513 | 144 | 4.5e-34 |
| CAG15012.1 | peptide\_synthetase,\_module\_7 | BGC0000441 | Teicoplanin\_biosynthetic\_gene\_cluster | 31 | 63.7621023513 | 144 | 4.5e-34 |
| ACM79810.1 | ZmaO | BGC0001059 | Zwittermycin\_A\_biosynthetic\_gene\_cluster | 31 | 50.7607192254 | 144 | 4.5e-34 |
| AAL15600.1 | SimH | BGC0000270 | Simocyclinone\_biosynthetic\_gene\_cluster | 33 | 47.1645919779 | 144 | 4.5e-34 |
| AAK06804.1 | putative\_peptide\_synthase\_SimD6 | BGC0001072 | Simocyclinone\_biosynthetic\_gene\_cluster | 33 | 47.1645919779 | 144 | 4.5e-34 |
| CAM02313.1 | putative\_non-ribosomal\_peptide\_synthetase | BGC0000349 | Erythrochelin\_biosynthetic\_gene\_cluster | 34 | 55.1867219917 | 144 | 5.9e-34 |
| BAF50711.1 | non\_ribosomal\_peptide\_synthetase\_for\_virginiamycin\_S | BGC0001116 | Virginiamycin\_biosynthetic\_gene\_cluster | 34 | 50.622406639 | 143 | 7.7e-34 |
| AAZ03554.1 | McnE | BGC0000332 | Cyanopeptolin\_biosynthetic\_gene\_cluster | 31 | 52.0055325035 | 143 | 1e-33 |
| AEP18656.1 | WAPS1 | BGC0000461 | WAP-8294A2\_(lotilibcin)\_biosynthetic\_gene\_cluster | 32 | 51.5905947441 | 143 | 1e-33 |
| AFH75328.1 | nonribosomal\_peptide\_synthetase | BGC0000398 | Orfamide\_biosynthetic\_gene\_cluster | 36 | 40.387275242 | 143 | 1e-33 |
| AFJ14794.1 | PlpE | BGC0000403 | Pelgipeptin\_biosynthetic\_gene\_cluster | 36 | 44.8132780083 | 143 | 1.3e-33 |
| CAE02631.1 | surfactin\_synthetase\_B | BGC0000433 | Surfactin\_biosynthetic\_gene\_cluster | 34 | 41.4937759336 | 143 | 1.3e-33 |
| CAE02630.1 | surfactin\_synthetase\_A | BGC0000433 | Surfactin\_biosynthetic\_gene\_cluster | 34 | 41.4937759336 | 143 | 1.3e-33 |
| CAJ96472.1 | Non-ribosomal\_peptide\_synthetase | BGC0000330 | Cupriachelin\_biosynthetic\_gene\_cluster | 34 | 49.377593361 | 143 | 1.3e-33 |
| ADD82940.1 | Bat2 | BGC0001099 | Kalimantacin\_/\_batumin\_biosynthetic\_gene\_cluster | 35 | 46.4730290456 | 143 | 1.3e-33 |
| AFJ23825.1 | WLIP\_synthetase\_B | BGC0000462\_c2 | WLIP\_biosynthetic\_gene\_cluster | 34 | 49.7925311203 | 143 | 1.3e-33 |
| AIS24862.1 | Dst18 | BGC0001147 | Distamycin\_biosynthetic\_gene\_cluster | 35 | 47.4412171508 | 143 | 1.3e-33 |
| CAC48360.1 | peptide\_synthetase | BGC0000311 | Balhimycin\_biosynthetic\_gene\_cluster | 35 | 44.8132780083 | 142 | 1.7e-33 |
| CUI25672.1 | Thiolactomycin\_Hybrid\_PKS-NRPS\_system | BGC0001353 | Thiolactomycin\_biosynthetic\_gene\_cluster | 34 | 46.6113416321 | 142 | 1.7e-33 |
| AHH53506.1 | non-ribosomal\_peptide\_synthetase | BGC0000439 | Taromycin\_biosynthetic\_gene\_cluster | 33 | 48.2710926694 | 142 | 1.7e-33 |
| ABD14711.1 | CesA | BGC0000320 | Cereulide\_biosynthetic\_gene\_cluster | 34 | 42.7385892116 | 142 | 2.2e-33 |
| AHZ34241.1 | CipD | BGC0001389 | Cichopeptin\_biosynthetic\_gene\_cluster | 34 | 44.6749654219 | 142 | 2.2e-33 |
| AEW31020.1 | plipastatin\_synthetase | BGC0000407 | Plipastatin\_biosynthetic\_gene\_cluster | 35 | 49.5159059474 | 142 | 2.2e-33 |
| AAN85506.1 | nonribosomal\_peptide\_synthetase | BGC0001101 | Leinamycin\_biosynthetic\_gene\_cluster | 33 | 50.4840940526 | 141 | 2.9e-33 |
| ABH06368.2 | MassB | BGC0000389 | Massetolide\_A\_biosynthetic\_gene\_cluster | 37 | 45.0899031812 | 141 | 2.9e-33 |
| CCM44336.1 | putative\_nonribosomal\_peptide\_synthetase | BGC0001056 | Zeamine\_biosynthetic\_gene\_cluster | 32 | 49.377593361 | 141 | 3.8e-33 |
| AAO62587.1 | peptide\_sythetase | BGC0001016 | Microcystin\_biosynthetic\_gene\_cluster | 31 | 50.3457814661 | 141 | 3.8e-33 |
| CAJ21198.2 | non-ribosomal\_peptide\_synthetase | BGC0000297 | Aeruginoside\_biosynthetic\_gene\_cluster | 33 | 47.9944674965 | 141 | 5e-33 |
| AKA59436.1 | non-ribosomal\_peptide\_synthetase | BGC0001202 | Landepoxcin\_biosynthetic\_gene\_cluster | 31 | 48.8243430152 | 141 | 5e-33 |
| BAP05596.1 | CalH | BGC0000967 | Calyculin\_biosynthetic\_gene\_cluster | 32 | 48.8243430152 | 140 | 6.5e-33 |
| ABP57748.1 | DepD | BGC0000993 | FK228\_biosynthetic\_gene\_cluster | 32 | 50.2074688797 | 140 | 6.5e-33 |
| AAY91419.3 | non-ribosomal\_peptide\_synthetase\_OfaA | BGC0000399 | Orfamide\_biosynthetic\_gene\_cluster | 33 | 49.7925311203 | 140 | 8.5e-33 |
| AAK81825.1 | peptide\_synthetase | BGC0000326 | Complestatin\_biosynthetic\_gene\_cluster | 33 | 43.9834024896 | 139 | 1.1e-32 |
| ADA82585.1 | hybrid\_trans-AT\_polyketide\_synthase\_-\_nonribosomal\_peptide\_synthetase | BGC0001110 | Psymberin\_biosynthetic\_gene\_cluster | 35 | 47.1645919779 | 139 | 1.1e-32 |
| ABI22132.1 | putative\_non-ribosomal\_peptide\_synthetase | BGC0000422 | Saframycin\_A\_biosynthetic\_gene\_cluster | 34 | 48.9626556017 | 139 | 1.5e-32 |
| AAF00961.1 | McyB | BGC0001017 | Microcystin\_biosynthetic\_gene\_cluster | 32 | 47.7178423237 | 139 | 1.9e-32 |
| CCJ67638.1 | TaaC | BGC0000447 | Tolaasin\_biosynthetic\_gene\_cluster | 33 | 58.5062240664 | 138 | 3.2e-32 |
| ABF87031.1 | non-ribosomal\_peptide\_synthetase/polyketide\_synthase | BGC0000393 | Myxoprincomide\_biosynthetic\_gene\_cluster | 32 | 52.1438450899 | 138 | 3.2e-32 |
| AHI59110.1 | locillomycin\_synthase\_C | BGC0001005 | Locillomycin\_biosynthetic\_gene\_cluster | 33 | 45.2282157676 | 138 | 4.2e-32 |
| AEF33078.1 | dimodular\_nonribosomal\_peptide\_synthetase | BGC0001039 | Pyridomycin\_biosynthetic\_gene\_cluster | 35 | 45.9197786999 | 137 | 5.5e-32 |
| AAY37653.1 | Amino\_acid\_adenylation | BGC0000437 | Syringomycin\_biosynthetic\_gene\_cluster | 35 | 44.6749654219 | 137 | 5.5e-32 |
| CAE14171.1 |  | BGC0000383 | Luminmycin\_biosynthetic\_gene\_cluster | 32 | 50.7607192254 | 137 | 7.2e-32 |
| ALG65314.1 | Cal22 | BGC0001297 | WS9326\_biosynthetic\_gene\_cluster | 33 | 49.7925311203 | 137 | 7.2e-32 |
| CCJ67647.1 | JagC | BGC0001127 | Jagaricin\_biosynthetic\_gene\_cluster | 32 | 48.5477178423 | 137 | 7.2e-32 |
| CBL93718.1 | NRPS\_didomain\_PCP-C | BGC0000360 | GE81112\_biosynthetic\_gene\_cluster | 31 | 52.0055325035 | 136 | 9.4e-32 |
| AAO62586.1 | peptide\_sythetase | BGC0001016 | Microcystin\_biosynthetic\_gene\_cluster | 33 | 45.9197786999 | 136 | 9.4e-32 |
| AAY32966.1 | DszC | BGC0001093 | Disorazole\_biosynthetic\_gene\_cluster | 30 | 53.9419087137 | 136 | 1.6e-31 |
| CBZ42146.1 | putative\_non-ribosomal\_peptide\_synthetase | BGC0001117 | Himastatin\_biosynthetic\_gene\_cluster | 32 | 67.6348547718 | 135 | 2.7e-31 |
| ACM68690.1 | AerG1 | BGC0000298 | Aeruginosin\_biosynthetic\_gene\_cluster | 30 | 46.1964038728 | 135 | 2.7e-31 |
| BAO84868.1 | putative\_non-ribosomal\_peptide\_synthetase | BGC0000414 | Quinocarcin\_biosynthetic\_gene\_cluster | 38 | 36.0995850622 | 134 | 3.6e-31 |
| AJV88375.1 | MfnC | BGC0001214 | Marformycins\_biosynthetic\_gene\_cluster | 36 | 42.8769017981 | 134 | 6.1e-31 |
| AEW31019.1 | plipastatin\_synthetase | BGC0000407 | Plipastatin\_biosynthetic\_gene\_cluster | 34 | 40.6639004149 | 133 | 8e-31 |
| CBL93720.1 | NRPS\_didomain\_PCP-A | BGC0000360 | GE81112\_biosynthetic\_gene\_cluster | 32 | 51.1756569848 | 133 | 1.4e-30 |
| ABC35522.1 | thiotemplate\_mechanism\_natural\_product\_synthetase | BGC0000186 | Thailandamide\_biosynthetic\_gene\_cluster | 33 | 50.8990318119 | 133 | 1.4e-30 |
| ABS74209.1 | FenA | BGC0001095 | Fengycin\_biosynthetic\_gene\_cluster | 34 | 40.8022130014 | 133 | 1.4e-30 |
| BAI63288.1 | putative\_non-ribosomal\_peptide\_synthetase | BGC0000434 | SW-163\_biosynthetic\_gene\_cluster | 35 | 45.7814661134 | 132 | 1.8e-30 |
| AAO62588.1 | peptide\_sythetase | BGC0001016 | Microcystin\_biosynthetic\_gene\_cluster | 31 | 46.887966805 | 131 | 3e-30 |
| ADJ63842.1 | non-ribosomal\_peptide\_synthetase\_protein | BGC0000424 | Serobactins\_biosynthetic\_gene\_cluster | 34 | 47.0262793914 | 131 | 3e-30 |
| EFL06865.1 | hypothetical\_protein | BGC0000300 | Amychelin\_biosynthetic\_gene\_cluster | 33 | 45.2282157676 | 131 | 3e-30 |
| AJW76710.1 | DsaH | BGC0001196 | Desotamide\_biosynthetic\_gene\_cluster | 34 | 47.1645919779 | 131 | 4e-30 |
| AID65222.1 | putative\_aspartate\_racemase | BGC0000335 | Cystomanamides\_biosynthetic\_gene\_cluster | 36 | 46.4730290456 | 131 | 4e-30 |
| BAI63283.1 | putative\_non-ribosomal\_peptide\_synthetase | BGC0000434 | SW-163\_biosynthetic\_gene\_cluster | 32 | 46.0580912863 | 131 | 5.2e-30 |
| sln9 | nonribosomal\_peptide\_synthetase | BGC0001230 | Salinamides\_biosynthetic\_gene\_cluster | 34 | 46.7496542185 | 130 | 8.8e-30 |
| AAG02364.1 | peptide\_synthetase\_NRPS2-1 | BGC0000963 | Bleomycin\_biosynthetic\_gene\_cluster | 33 | 51.0373443983 | 130 | 8.8e-30 |
| CAD29799.1 | microcystin\_synthetase | BGC0001015 | Microcystin\_biosynthetic\_gene\_cluster | 32 | 46.3347164592 | 129 | 1.2e-29 |
| BAH33409.1 | putative\_non-ribosomal\_peptide\_synthetase | BGC0000371 | Heterobactin\_biosynthetic\_gene\_cluster | 36 | 41.7704011065 | 129 | 1.5e-29 |
| CAG29032.1 | nonribosomal\_peptide\_synthetase\_(modules\_3\_to\_6) | BGC0001023 | Myxochromide\_biosynthetic\_gene\_cluster | 31 | 50.4840940526 | 129 | 1.5e-29 |
| AAF00960.1 | McyA | BGC0001017 | Microcystin\_biosynthetic\_gene\_cluster | 33 | 41.6320885201 | 129 | 2e-29 |
| AAZ23076.1 | peptide\_synthetase | BGC0000291 | A54145\_biosynthetic\_gene\_cluster | 31 | 49.377593361 | 128 | 2.6e-29 |
| AGC09519.1 | AMP-dependent\_synthetase/ligase | BGC0001183 | Lobophorin\_biosynthetic\_gene\_cluster | 32 | 46.7496542185 | 128 | 2.6e-29 |
| ABV79986.1 | ApnB | BGC0000301 | Anabaenopeptin\_biosynthetic\_gene\_cluster | 33 | 42.6002766252 | 128 | 4.4e-29 |
| AHH25592.1 | NRPS | BGC0000957 | Ansatrienin\_(mycotrienin)\_biosynthetic\_gene\_cluster | 34 | 44.1217150761 | 127 | 7.5e-29 |
| ABA70582.1 | alpha-aminoadypil-cysteinyl-valine\_synthetase | BGC0000404 | Penicillin\_biosynthetic\_gene\_cluster | 30 | 50.7607192254 | 127 | 7.5e-29 |
| ABR12615.1 | ACV\_synthetase | BGC0000405 | Penicillin\_biosynthetic\_gene\_cluster | 30 | 50.7607192254 | 127 | 7.5e-29 |
| AAS92545.1 | SirP | BGC0001044 | Sirodesmin\_biosynthetic\_gene\_cluster | 32 | 48.132780083 | 126 | 9.8e-29 |
| WP\_028678149.1 | non-ribosomal\_peptide\_synthetase | BGC0001228 | Retimycin\_biosynthetic\_gene\_cluster | 32 | 45.0899031812 | 125 | 2.2e-28 |
| AHD05614.1 | putative\_non-ribosomal\_peptide\_ligase/\_polyketide\_synthase\_hybrid | BGC0001033 | Paenilamicin\_biosynthetic\_gene\_cluster | 28 | 55.0484094053 | 125 | 2.2e-28 |
| AFK57219.1 | DidH | BGC0000985 | Didemnin\_biosynthetic\_gene\_cluster | 30 | 73.8589211618 | 125 | 2.8e-28 |
| AID65224.1 | nonribosomal\_peptide\_synthetase | BGC0000335 | Cystomanamides\_biosynthetic\_gene\_cluster | 32 | 41.6320885201 | 125 | 2.8e-28 |
| AAT70101.1 | CurF | BGC0001165 | Curacin\_biosynthetic\_gene\_cluster | 28 | 50.3457814661 | 124 | 4.9e-28 |
| AAP92491.1 | nonribosomal\_peptide\_synthetase | BGC0000458 | Viomycin\_biosynthetic\_gene\_cluster | 32 | 47.1645919779 | 124 | 4.9e-28 |
| AEE88284.1 | CurF | BGC0000976 | Curacin\_biosynthetic\_gene\_cluster | 28 | 50.3457814661 | 124 | 4.9e-28 |
| AID65225.1 | nonribosomal\_peptide\_synthetase | BGC0000335 | Cystomanamides\_biosynthetic\_gene\_cluster | 32 | 41.6320885201 | 124 | 6.3e-28 |
| CDG76959.1 | non-ribosomal\_peptide\_synthetase,\_terminal\_component | BGC0000446 | Tilivalline\_biosynthetic\_gene\_cluster | 30 | 53.5269709544 | 123 | 1.1e-27 |
| WP\_051700105.1 | non-ribosomal\_peptide\_synthetase | BGC0001368 | Tambromycin\_biosynthetic\_gene\_cluster | 32 | 47.1645919779 | 123 | 1.1e-27 |
| ABI26078.1 | OciB | BGC0000331 | Cyanopeptin\_biosynthetic\_gene\_cluster | 42 | 25.7261410788 | 123 | 1.4e-27 |
| ALV82447.1 | putative\_non-ribosomal\_peptide\_synthetase | BGC0001382 | Svaricin\_biosynthetic\_gene\_cluster | 31 | 49.2392807746 | 122 | 2.4e-27 |
| AKA59447.1 | non-ribosomal\_peptide\_synthetase | BGC0001203 | Clarexpoxcin\_biosynthetic\_gene\_cluster | 31 | 50.0691562932 | 122 | 2.4e-27 |
| AAP92497.1 | nonribosomal\_peptide\_synthetase | BGC0000458 | Viomycin\_biosynthetic\_gene\_cluster | 32 | 45.9197786999 | 121 | 3.1e-27 |
| CAD89774.1 | MelC\_protein | BGC0001010 | Melithiazol\_biosynthetic\_gene\_cluster | 30 | 49.1009681881 | 121 | 3.1e-27 |
| BAN91483.1 | putative\_Aculeacin\_A\_nonribosomal\_peptide\_synthase | BGC0001220 | Aculeacin\_A\_biosynthetic\_gene\_cluster | 30 | 46.0580912863 | 120 | 7e-27 |
| ADH04680.1 | hybrid\_polyketide\_synthase/non-ribosomal\_peptide\_synthetase | BGC0001344 | Tubulysin\_biosynthetic\_gene\_cluster | 29 | 49.377593361 | 120 | 7e-27 |
| ABR67744.1 | CmnA | BGC0000316 | Capreomycin\_biosynthetic\_gene\_cluster | 38 | 32.2268326418 | 120 | 9.2e-27 |
| ADZ45324.1 | non-ribosomal\_peptide\_synthetase | BGC0001020 | Muraymycin\_biosynthetic\_gene\_cluster | 32 | 50.622406639 | 119 | 1.6e-26 |
| CAD70194.1 | non-ribosomal\_peptide\_synthetase | BGC0001047 | Syringolin\_A\_biosynthetic\_gene\_cluster | 29 | 44.9515905947 | 119 | 1.6e-26 |
| EJK79842.1 | amino\_acid\_adenylation\_enzyme/thioester\_reductase\_family\_protein | BGC0000436 | Syringolin\_A\_biosynthetic\_gene\_cluster | 29 | 44.8132780083 | 118 | 2.7e-26 |
| CAD70195.1 | non-ribosomal\_peptide\_synthetase | BGC0001047 | Syringolin\_A\_biosynthetic\_gene\_cluster | 31 | 46.7496542185 | 118 | 4.5e-26 |
| AAT09805.1 | NocB | BGC0000395 | Nocardicin\_A\_biosynthetic\_gene\_cluster | 32 | 51.7289073306 | 118 | 4.5e-26 |
| BAO73252.1 | non-ribosomal\_peptide\_synthetase | BGC0000456 | Verlamelin\_biosynthetic\_gene\_cluster | 31 | 48.9626556017 | 117 | 7.7e-26 |
| AAW03326.1 | CtaC | BGC0000982 | Cystothiazole\_A\_biosynthetic\_gene\_cluster | 29 | 49.1009681881 | 116 | 1e-25 |
| AAC06346.1 | bacitracin\_synthetase\_1 | BGC0000310 | Bacitracin\_biosynthetic\_gene\_cluster | 29 | 45.0899031812 | 116 | 1e-25 |
| BAD55613.1 | putative\_non-ribosomal\_peptide\_synthetase | BGC0001027 | Nocobactin\_NA\_biosynthetic\_gene\_cluster | 31 | 52.6970954357 | 116 | 1.3e-25 |
| CAJ88610.1 | putative\_non-ribosomal\_peptide\_synthetase | BGC0000327 | Congocidine\_biosynthetic\_gene\_cluster | 32 | 47.3029045643 | 116 | 1.7e-25 |
| ADG27359.1 | peptide\_synthetase | BGC0000296 | Actinomycin\_biosynthetic\_gene\_cluster | 41 | 29.18395574 | 115 | 2.9e-25 |
| ACZ66258.1 | APS1 | BGC0000304 | Apicidin\_biosynthetic\_gene\_cluster | 30 | 46.4730290456 | 112 | 1.9e-24 |
| AAZ03552.1 | McnC | BGC0000332 | Cyanopeptolin\_biosynthetic\_gene\_cluster | 43 | 20.4702627939 | 112 | 2.5e-24 |
| ADQ74618.1 | amino\_acid\_adenylation\_protein | BGC0000921 | Promysalin\_biosynthetic\_gene\_cluster | 33 | 41.9087136929 | 111 | 3.3e-24 |
| ABV56594.1 | KtzN | BGC0000378 | Kutznerides\_biosynthetic\_gene\_cluster | 33 | 37.0677731674 | 111 | 3.3e-24 |
| ABR67750.1 | CmnG | BGC0000316 | Capreomycin\_biosynthetic\_gene\_cluster | 30 | 46.0580912863 | 111 | 4.3e-24 |
| AHB82058.1 | non\_ribosomal\_peptide\_synthetase | BGC0001019 | Microsclerodermins\_biosynthetic\_gene\_cluster | 36 | 31.5352697095 | 111 | 4.3e-24 |
| AMK48225.1 | nonribosomal\_peptide\_synthetase | BGC0001351 | Cahuitamycins\_biosynthetic\_gene\_cluster | 32 | 48.4094052559 | 111 | 4.3e-24 |
| AGS06887.1 | mixed\_type\_I\_polyketide\_synthase/non-ribosomal\_peptide\_synthetase,\_PedF-like\_protein | BGC0001092\_c2 | Diaphorin\_biosynthetic\_gene\_cluster | 30 | 47.9944674965 | 111 | 5.6e-24 |
| AEO14743.1 | NdaA | BGC0000396 | Nodularin\_biosynthetic\_gene\_cluster | 34 | 29.7372060858 | 110 | 7.3e-24 |
| KPN90374.1 | NunB1 | BGC0001416 | Nunapeptin\_/\_nunamycin\_biosynthetic\_gene\_cluster | 32 | 46.3347164592 | 110 | 7.3e-24 |
| AIW82277.1 | PuwA | BGC0001125 | Puwainaphycins\_biosynthetic\_gene\_cluster | 39 | 27.3858921162 | 109 | 2.1e-23 |
| EFL06864.1 | non-ribosomal\_peptide\_synthetase | BGC0000300 | Amychelin\_biosynthetic\_gene\_cluster | 31 | 43.5684647303 | 108 | 2.8e-23 |
| CAD29797.1 | peptide\_synthetase | BGC0001015 | Microcystin\_biosynthetic\_gene\_cluster | 31 | 33.6099585062 | 108 | 4.7e-23 |
| CAJ34370.1 | NRPS\_protein | BGC0000445 | Thiocoraline\_biosynthetic\_gene\_cluster | 33 | 34.7164591978 | 104 | 5.2e-22 |
| AFK57215.1 | DidD | BGC0000985 | Didemnin\_biosynthetic\_gene\_cluster | 37 | 28.0774550484 | 104 | 6.8e-22 |
| CAG29031.1 | nonribosomal\_peptide\_synthetase\_(modules\_1\_and\_2) | BGC0001023 | Myxochromide\_biosynthetic\_gene\_cluster | 37 | 25.3112033195 | 103 | 8.9e-22 |
| AAQ93484.1 | CmaA | BGC0000328 | Coronatine\_biosynthetic\_gene\_cluster | 32 | 42.6002766252 | 102 | 1.5e-21 |
| CAQ18839.1 | hybrid\_polyketide\_synthase/nonribosomal\_polypetide\_synthetase | BGC0000954 | Ajudazol\_biosynthetic\_gene\_cluster | 37 | 28.0774550484 | 102 | 2e-21 |
| WP\_028678148.1 | non-ribosomal\_peptide\_synthetase | BGC0001228 | Retimycin\_biosynthetic\_gene\_cluster | 34 | 30.5670816044 | 100 | 7.5e-21 |
| CAP93139.1 |  | BGC0000357 | Fungisporin\_biosynthetic\_gene\_cluster | 28 | 52.4204702628 | 100 | 7.5e-21 |
| ACN39728.1 | SibE | BGC0000428 | Sibiromycin\_biosynthetic\_gene\_cluster | 29 | 41.9087136929 | 100 | 9.8e-21 |
| WP\_051700122.1 | non-ribosomal\_peptide\_synthetase | BGC0001368 | Tambromycin\_biosynthetic\_gene\_cluster | 31 | 46.887966805 | 99 | 1.3e-20 |
| BAO66533.1 | nonribosomal\_peptide\_synthase | BGC0000042 | Cremimycin\_biosynthetic\_gene\_cluster | 32 | 36.5145228216 | 95 | 3.2e-19 |
| CAJ34375.1 | NRPS | BGC0000445 | Thiocoraline\_biosynthetic\_gene\_cluster | 37 | 24.4813278008 | 95 | 3.2e-19 |
| OAQ83772.1 | nonribosomal\_peptide\_synthase | BGC0001358 | Leucinostatins\_biosynthetic\_gene\_cluster | 29 | 46.887966805 | 94 | 4.1e-19 |
| BAR73008.1 | putative\_ATP-dependent\_b-aminoacyl-ACP\_synthetase | BGC0001194 | Hitachimycin\_biosynthetic\_gene\_cluster | 31 | 35.8229598893 | 94 | 7e-19 |
| AGZ15455.1 | non-ribosomal\_peptide\_synthetase | BGC0001036 | Polyoxypeptin\_biosynthetic\_gene\_cluster | 35 | 36.2378976487 | 93 | 9.2e-19 |
| CBW45640.1 | PII\_peptide\_synthetase | BGC0000952 | Pristinamycin\_biosynthetic\_gene\_cluster | 30 | 35.40802213 | 92 | 2e-18 |
| AGI61652.1 | long\_chain\_fatty\_acid\_CoA\_ligase | BGC0000095\_c2 | Micromonolactam\_biosynthetic\_gene\_cluster | 30 | 36.5145228216 | 91 | 3.5e-18 |
| ALA09365.1 | AMP-dependent\_synthetase\_and\_ligase | BGC0001303 | Lobosamide\_biosynthetic\_gene\_cluster | 32 | 35.1313969571 | 91 | 4.6e-18 |
| BAO84864.1 | putative\_non-ribosomal\_peptide\_synthetase | BGC0000414 | Quinocarcin\_biosynthetic\_gene\_cluster | 28 | 39.9723374827 | 90 | 1e-17 |
| ABP55217.1 | AMP-dependent\_synthetase\_and\_ligase | BGC0000142 | Salinilactam\_biosynthetic\_gene\_cluster | 30 | 35.1313969571 | 88 | 3e-17 |
| ABX71111.1 | Lct28 | BGC0000238 | Lactonamycin\_biosynthetic\_gene\_cluster | 34 | 39.5573997234 | 87 | 5e-17 |
| ACO94461.1 | putative\_AMP-dependent\_acyl-CoA\_synthetase/ligase | BGC0000029 | BE-14106\_biosynthetic\_gene\_cluster | 30 | 36.7911479945 | 85 | 2.5e-16 |
| CBF87069.1 | nonribosomal\_peptide\_synthase,\_putative\_(Eurofung) | BGC0001290 | Emericellamide\_A\_/\_emericellamide\_B\_biosynthetic\_gene\_cluster | 27 | 47.1645919779 | 84 | 4.3e-16 |
| CCB53264.1 | non-ribosomal\_peptide\_synthetase | BGC0001393 | Lugdunin\_biosynthetic\_gene\_cluster | 27 | 35.2697095436 | 79 | 2.3e-14 |
| ABB69081.1 | putative\_L-prolyl-AMP\_ligase | BGC0000260 | Prodigiosin\_biosynthetic\_gene\_cluster | 34 | 37.0677731674 | 78 | 3.1e-14 |
| ADH04681.1 | non-ribosomal\_peptide\_synthetase | BGC0001344 | Tubulysin\_biosynthetic\_gene\_cluster | 28 | 41.4937759336 | 78 | 4e-14 |
| CCB53266.1 | non-ribosomal\_peptide\_synthetase | BGC0001393 | Lugdunin\_biosynthetic\_gene\_cluster | 23 | 47.3029045643 | 70 | 1.1e-11 |
| AAN32981.1 | BarG | BGC0000962 | Barbamide\_biosynthetic\_gene\_cluster | 32 | 20.7468879668 | 69 | 1.4e-11 |
| ABW00331.1 | amino\_acid\_adenylation\_domain | BGC0000333 | Cyclomarin\_biosynthetic\_gene\_cluster | 30 | 24.2047026279 | 69 | 1.4e-11 |
| AFO85453.1 | non-ribosomal\_peptide\_synthetase | BGC0000391 | Methylpendolmycin/pendolmycin\_biosynthetic\_gene\_cluster | 33 | 24.2047026279 | 67 | 7e-11 |
| BAO98812.1 | putative\_nonribosomal\_peptide\_synthetase | BGC0001002 | Jawsamycin\_biosynthetic\_gene\_cluster | 28 | 35.1313969571 | 67 | 9.2e-11 |
| AGA37269.1 | NRPS | BGC0000819 | Paraherquamide\_biosynthetic\_gene\_cluster | 27 | 37.4827109267 | 63 | 1.3e-09 |
| AGZ15460.1 | putative\_non-ribosomal\_peptide\_synthetase | BGC0001036 | Polyoxypeptin\_biosynthetic\_gene\_cluster | 32 | 20.8852005533 | 62 | 3e-09 |
| AAC83657.1 | pyochelin\_synthetase | BGC0000412 | Pyochelin\_biosynthetic\_gene\_cluster | 28 | 25.1728907331 | 61 | 5e-09 |
| AJV88377.1 | MfnE | BGC0001214 | Marformycins\_biosynthetic\_gene\_cluster | 28 | 20.4702627939 | 61 | 5e-09 |
| AHB38497.1 | EpxD | BGC0000346 | Epoxomicin\_biosynthetic\_gene\_cluster | 30 | 21.9917012448 | 60 | 8.6e-09 |
| CAF05650.1 | TubE\_protein | BGC0001053 | Tubulysin\_biosynthetic\_gene\_cluster | 25 | 41.0788381743 | 57 | 7.3e-08 |
| AAF19812.1 | MtaD | BGC0001024 | Myxothiazol\_biosynthetic\_gene\_cluster | 29 | 21.3001383126 | 54 | 8.1e-07 |
| CBW45647.1 | PI\_synthetase\_3\_and\_4 | BGC0000952 | Pristinamycin\_biosynthetic\_gene\_cluster | 30 | 20.3319502075 | 52 | 1.8e-06 |
| AET98914.1 | putative\_AMP-binding\_ligase | BGC0000415 | Quinomycin\_biosynthetic\_gene\_cluster | 26 | 32.6417704011 | 51 | 5.2e-06 |
